# Dietary Oncopharmacognosy as a Crosswalk between Precision Oncology and Precision Nutrition

**DOI:** 10.3390/nu15092219

**Published:** 2023-05-08

**Authors:** Henry J. Thompson, Tymofiy Lutsiv, John N. McGinley, Hisham Hussan, Mary C. Playdon

**Affiliations:** 1 Cancer Prevention Laboratory, Colorado State University, Fort Collins, CO 80523, USA; tymofiy.lutsiv@colostate.edu (T.L.); john.mcginley@colostate.edu (J.N.M.); 2 Cell and Molecular Biology Graduate Program, Colorado State University, Fort Collins, CO 80523, USA; 3 Department of Internal Medicine, University of California Davis, Sacramento, CA 95817, USA; hhussan@ucdavis.edu; 4 Department of Nutrition and Integrative Physiology, University of Utah, Salt Lake City, UT 84112, USA; mary.playdon@hci.utah.edu

**Keywords:** precision oncology, precision nutrition, bioactive food components, cancer prevention and control, diet and nutrition, dietary oncopharmacognosy, pharmacology, natural products

## Abstract

While diet and nutrition are modifiable risk factors for many chronic and infectious diseases, their role in cancer prevention and control remains under investigation. The lack of clarity of some diet–cancer relationships reflects the ongoing debate about the relative contribution of genetic factors, environmental exposures, and replicative errors in stem cell division as determinate drivers of cancer risk. In addition, dietary guidance has often been based upon research assuming that the effects of diet and nutrition on carcinogenesis would be uniform across populations and for various tumor types arising in a specific organ, i.e., that one size fits all. Herein, we present a paradigm for investigating precision dietary patterns that leverages the approaches that led to successful small-molecule inhibitors in cancer treatment, namely understanding the pharmacokinetics and pharmacodynamics of small molecules for targeting carcinogenic mechanisms. We challenge the scientific community to refine the paradigm presented and to conduct proof-in-concept experiments that integrate existing knowledge (drug development, natural products, and the food metabolome) with developments in artificial intelligence to design and then test dietary patterns predicted to elicit drug-like effects on target tissues for cancer prevention and control. We refer to this precision approach as dietary oncopharmacognosy and envision it as the crosswalk between the currently defined fields of precision oncology and precision nutrition with the goal of reducing cancer deaths.

## 1. Introduction

Given the impact of diet and nutrition on many chronic and infectious disease processes, an expectation has existed for decades that similar effects would be observed for cancer [1,2]. Yet, despite a global effort to identify those linkages, the literature supporting diet’s role in cancer development and progression has inconsistencies [3,4,5]. In addressing this conundrum, one recent *Nature* perspective calls for higher quality research in this arena [6]. This is undoubtedly needed. Similar to the World Health Organization’s approach to addressing the relationship between obesity and cancer [7], the area of diet and nutrition in cancer requires a bottom-up (mechanistic) as well as a top-down (population to clinical studies) process to reframe key questions and identify high probability causal relationships at the interface of precision oncology and precision nutrition that can be translated to precision public health. Despite small-molecule inhibitor pharmaceuticals becoming a mainstay of cancer treatment, the paradigm has failed to seize the opportunity to leverage culinary medicine and exposure to food-derived small molecules to improve patient outcomes. Herein, we re-introduce two concepts from the mid-1970s: (1) targeting the entire process of carcinogenesis for treatment and (2) suppressing, inhibiting, and reversing any phase of the disease process from the generation of a cancer-initiated stem cell to cancer specific death after a cancer diagnosis [8,9]. The success of targeted treatment with small-molecule inhibitors of protein kinases, apoptosis evasion, immune suppression, and angiogenesis induction in reducing cancer deaths provides a framework for re-examining the role of diet and nutrition in cancer prevention. We leverage the pioneering science that identified the origins of cancer driving mutations and emerging hallmarks of cancer to identify targets for precision nutrition: (1) the protein products of the genetic drivers of carcinogenesis [10,11,12,13] and (2) tissue-specific effects on the carcinogenic potential of cells bearing driver mutations within that tissue, i.e., “field effects” [14,15,16]. This review will focus on the potential effects of diet and nutrition on drivers of carcinogenesis. 

## 2. Definitions

In addition to re-introducing the purpose of treating carcinogenesis as the prevention of death from cancer, definitions of precision public health, precision medicine, precision oncology, and precision nutrition, and subdisciplines therein, are important to delineate. At their core, several overarching themes distinguish precision approaches in each of these disciplines from their conventional counterparts. Two of those themes are (1) that one size does not fit all, and (2) that genetic analyses and those of other omics platforms and behavioral assessment paradigms are used to predict and triage populations and individuals into plans of action that are most likely to work for them.

*Precision medicine* is an approach to disease treatment and prevention that considers individual variability in genes, environment, and lifestyle for each person [17]. This approach allows physicians to predict more accurately which prevention and treatment strategies for a particular disease will work in specific groups of people. It is in contrast to a one-size-fits-all approach, in which disease prevention and treatment strategies are developed for the average person (i.e., population-based health guidance), with less consideration for the differences among individuals.

*Precision public health* is defined as the use of data and evidence to tailor interventions to the characteristics of a single population [18]. It differs from precision medicine in terms of its focus on populations and limited use of human genomics data, although this is changing as progress is made in molecular epidemiology.

*Precision oncology* is defined as cancer diagnosis, prognosis, prevention and/or treatment tailored specifically to the individual patient based on the patient’s genetic and/or molecular profile [19]. Exemplar approaches include targeted immunotherapy and mechanism-based therapies targeting specific cellular signaling pathways.

*Oncopharmacology* is the development and testing of drugs and their interactions with cancer cells [20].

*Precision nutrition* is an approach to developing comprehensive and dynamic nutritional recommendations based on individual variables, including genetics, microbiome, metabolic profile, health status, physical activity, dietary pattern, food environment, and socioeconomic and psychosocial characteristics [21]. In some respects, precision nutrition can be considered a subdiscipline of precision public health.

*Precision onconutrition* is the use of specific nutrients and dietary factors to enhance cancer treatment efficacy and to improve the prognosis for long-term survival. Precision onconutrition requires integration of an understanding of nutrient metabolism with knowledge of the signaling pathways characteristic of each molecular subtype of cancer which can be targeted to improve treatment and control efficacies [22].

*Dietary oncopharmacognosy*, as defined herein, identifies bioactive small molecules derived from dietary/food patterns (i.e., the collection of foods regularly consumed in a population) with cancer-relevant effects. The collection of bioactive small molecules linked to specific dietary patterns is either directly assimilated by the host during the process of food digestion or metabolized/modified by the microorganisms in the gut and thereafter exerts local and systemic effects.

## 3. The Top-Down Approach: Dietary Patterns

Under the backdrop that single nutrients are linked to single diseases or deficiency syndromes, e.g., vitamin C and scurvy, vitamin D and rickets, expectations were high that cancer could be prevented by a similar single nutrient paradigm as reflected by the content of one of the first comprehensive reviews of the topic in 1982 [23]. However, 50 years of investigation have generally failed to affirm these expectations [24]. In fact, the last fifteen years have witnessed an important airing of concern that diet and nutrition may not significantly impact the carcinogenic process based on the reductionist approach exemplified by single nutrient-based hypothesis testing [25,26,27,28], and in some specific cases, this approach could be harmful, e.g., vitamin A and lung cancer in smokers [29]. However, over the past decade, the single nutrient reductionist approach has been supplanted with a focus on how foods are typically consumed, i.e., dietary/food patterns, and with the analysis of cancer risk and cancer mortality when various dietary patterns are consumed [30,31,32]. This is based on the recognition that (1) individuals consume foods rather than only nutrients, (2) each food is composed of hundreds to thousands of chemicals (phytochemicals if the foods are of plant origin), many of which have bioactivity (bioactive food components), and (3) that foods eaten throughout a day can have synergistic, antagonistic, or agnostic actions that collectively may exert effects on the carcinogenic process.

The movement in the field from single nutrients to dietary/food patterns to understand the mechanistic links between diet and cancer is significant, but most studies use host systemic markers, e.g., circulating biomarkers, such as C-reactive protein for inflammation, insulin and C-peptide for insulin resistance, rather than tissue-specific markers to infer candidate mechanisms [33]. This approach may be too non-specific and lack sensitivity of the magnitude that has led to clinically effective mechanism-based small-molecule inhibitors used in cancer treatment. These well-tolerated, small-molecule pharmaceutical inhibitors that focus on specific dysregulated cellular targets have shown success with limited or no effects on host systemic biomarkers [34,35,36]. Yet, a food-derived bioactive-small-molecule approach to cancer prevention and control is lacking. A translational (i.e., both bottom-up and top-down) approach coupled with an understanding of food from a natural products/drug development perspective has the potential to unveil culinary medicine dietary approaches that elicit drug-like effects on target tissues for cancer prevention and control in specific populations. In making this statement, we emphasize that we are not proposing to make drugs from food molecules but rather are recommending the formulation of precision diets that have effects parallel to targeted therapies and that complement those treatment approaches when they are initiated.

## 4. Bottom-Up: Natural Products from Food

A simple definition of “natural product” is any molecule produced by a living organism [37]. While the data are limited, recent large meta-analyses indicate that some natural products of animal origin enhance cancer development [38]. In contrast, natural products in foods of plant origin commonly consumed in the diet have a greater propensity for inhibiting the carcinogenic process [39]. Since the focus of this narrative review is on the prevention and control of cancer, we will focus on foods that originate from plants, recognizing that a reduction in animal product consumption could also be protective.

Plants synthesize chemicals (therefore referred to as phytochemicals) that are structural or involved in metabolic processes such as reproduction, cellular defense, and cell signaling within a plant and among plants [40,41]. Generally, phytochemicals are categorized as either primary or secondary metabolites. Primary metabolites include carbohydrates, fats, proteins, and nucleic acids. Plant secondary metabolites can be classified into four major classes: terpenoids, phenolic compounds, alkaloids, and sulfur-containing compounds. They have low molecular weight (<1500 dalton) and are estimated to include over 10,000 distinct chemicals [40,41]. Primary metabolites are a source of macronutrients for humans, and secondary metabolites are a source of micronutrients (vitamins and minerals) and bioactive food components.

## 5. From Food Patterns and Their Bioactive Food Components to Precision Oncology

Oncopharmacology is the developing and testing drugs and their interactions with cancer cells [22]. Analogously, pharmacognosy in oncology (which we term *dietary oncopharmacognosy*) identifies bioactive small molecules derived from dietary/food patterns, i.e., the collection of foods and beverages regularly consumed in a population, with cancer-relevant effects. The collection of bioactive small molecules linked to specific dietary patterns is either directly assimilated by the host during the process of food digestion or metabolized/modified by the microorganisms in the gut and thereafter exerts local and systemic effects [42,43]. Integration of pharmacognosy with dietary pattern research gives a novel perspective when juxtaposed with an increasing portfolio of clinically effective drugs that are proven small-molecule inhibitors of various aspects of the carcinogenic process [44]. In fact, many of the drugs currently in use have their origins in natural products research, albeit the natural analogs have lower target specificity and affinity. Of particular interest are small-molecule inhibitors of specific types of protein kinases that are dysregulated throughout the carcinogenic process and that vary by cancer type, as well as small molecules that permit reactivation of apoptosis, block angiogenesis, or inhibit the misregulation of immune checkpoints [34,45,46]. In the clinical and public health space, food pharmacies are an emerging model designed to improve access to healthful foods using food prescriptions with the goal of preventing and managing disease [47]. Food pharmacies are a potential future avenue for disseminating dietary prescriptions (healthful groceries and/or medically tailored meals, MTM) designed to target carcinogenic processes with food-derived bioactive molecules [48]. To our knowledge, there has not been a systematic interrogation of the bioactive food component repertoire, which is also referred to as *the food metabolome*, provided via the regular consumption of a precision food pattern that targets multiple facets of cancer hallmark events. It is anticipated that an effective chemical array will delay, inhibit, or reverse cancer initiation, promotion, or progression in a manner that reduces cancer deaths.

## 6. A Mechanistic Foundation

As noted in the Introduction section, a key distinction of the approach described herein is that it is based on demonstrated clinical efficacy in the use of small-molecule inhibitors in cancer treatment [49,50,51]. This explains a predominant focus on inhibitors of protein kinases [44,52,53]. However, other critical, dysregulated cellular processes involved in carcinogenesis are being effectively targeted in cancer treatment, although the number of small-molecule drugs in clinical use is less extensive. What is key for protein kinases and for inhibitors directed at apoptosis, angiogenesis, and immune checkpoint regulation is that misregulation of these processes is stoichiometric, i.e., determined by quantitative balance that exists relative to positive and negative regulators of the process [54]. An effective small-molecule inhibitor shifts the balance toward normal cellular function in each case. Emerging evidence that many targets have effects across multiple cellular processes will provide a basis for delineating synergistic effects.

### 6.1. Protein Kinases

The signature feature of cancer initiation, i.e., the occurrence of a driver mutation in a cell, is that it confers a selective growth advantage on that cell that results in an expanding clone of mutated cells [10,15,55,56,57]. In stating that a selective growth advantage occurs, it simply means that the rate of cell birth exceeds the rate of cell death. It is estimated that the magnitude of this disequilibrium is 0.06%, i.e., six excess births for every ten thousand cell divisions, sufficient to give rise to a clinically detectable tumor over the 15- to 20-year latency period associated with the development of cancer [58]. The occurrence of driver mutations of environmental or replicative origin is considered a stochastic process; however, most evidence points to the need for multiple driver mutations for a transformed cell to become a clinically detectable cancer [59,60]. Cancer results from mutations in various combinations in the genes that control three core functions (i.e., proliferation, apoptosis, and angiogenesis) via 12 signaling pathways. Driver mutation genes are generally broken into two categories: oncogenes and tumor suppressor genes that produce proteins with different activity [15,55,56]. Most proto-oncogenes code for protein kinases, and the mutation of the gene converts the proto-oncogene into a driver mutation gene resulting in a constitutively hyperactive protein. The amount of hyperactive protein in the cell must exceed some threshold for the cell to manifest an altered, neoplastic phenotype [61]. *This is the origin of stoichiometry for cancer prevention and control*. Based on this knowledge, specific inhibitors can target specific oncogenic protein kinases, with demonstrated clinical efficacy in slowing cancer progression and even in eliminating cancer cells. More broadly, the level of activity can also be controlled by inducing protein degradation or epigenetic regulation of gene expression. However, many of the driver genes are tumor suppressors, where mutation results in loss of function [55]. Initially, it was thought that defects in protein expression of this type could not be targeted. However, with continued progress in understanding downstream events usually regulated by tumor suppressors, it has been demonstrated that loss of this function results in the hyperactivation of protein kinases. As discussed above, these protein kinases are druggable targets [44,62,63].

These considerations have important implications for developing a mechanism-based understanding of how an effective precision dietary pattern could inhibit the development of cancer. Specifically, in considering dietary oncopharmacognosy, it provides a specific set of target kinases that dietary bioactive food components inhibit, which provides a basis for pharmacodynamic modeling. In combination with pharmacokinetic modeling of phytochemical mixtures, whole body PK/PD will identify not only the basis of protective activity of specific dietary patterns but also provide the understanding of the interchangeability of foods within a dietary pattern and the number of foods and the frequency of consumption required to suppress the oncogenic activity of various kinases sustainably. Moreover, a basis is provided for understanding the multiplicative and synergistic effects rendered by a dietary pattern relative to its individual components based on the pattern of protein kinases that are inhibited as well as whether the dietary inhibitors preferentially target kinases in specific pathways in which driver mutations are known to occur. Finally, while the specificity of kinase inhibitors is critical in cancer treatment [62], the likely lower specificity of dietary chemicals may increase the power of diet to affect multiple loci during the development of cancer. This would effectively suppress the disease process by reducing the accumulation of oncogenic protein activity such that the critical intracellular threshold is never reached.

There has been extensive mining of natural product resources for novel protein kinase inhibitors using kinome inhibition assays [44,64]. This provides a crosswalk to the foods comprising a dietary pattern through the lens of their botanical classification. Specifically, we have proposed that the chemical diversity of a food pattern can be predicted by understanding the botanical origin of those foods [65]. More recently, we have used the same botanical approach to illustrate that small-molecule inhibitors, e.g., protein kinase inhibitors, are found in the same botanical families from which human foods are derived and that the nature of the protein kinases inhibited varies by botanical family [66]. At this juncture, we are unaware of the use of this approach either in assessing the activity of effective dietary patterns or as the rationale for formulating precision dietary patterns based on their demonstrated activity in inhibiting oncogenic protein kinases.

### 6.2. Apoptosis

One of the hallmarks of human cancers is the intrinsic or acquired resistance to apoptosis [15,55,56]. Whether a cellular response to ensure the cell’s survival upon exposure to stressful stimuli or a consequence of oncogenic protein kinase transformation, apoptosis resistance contributes to carcinogenesis, tumor progression, and treatment resistance. Relative to refractoriness to treatment, most current anti-cancer therapies, including chemotherapy as well as radio- and immunotherapies, primarily act by activating cell death pathways, including apoptosis, in cancer cells. Examples of targeting both the intrinsic (inhibitors of BCL2, MCL, and IAP/survivin) and extrinsic pathways (death receptor agonists) that regulate apoptosis have been published, and natural products that modulate apoptosis have recently been reviewed [67,68]. A specific paradigm-shifting example is the use of venetaclax, a small molecule BH3 mimetic, as a sole therapy for the treatment of chronic lymphocytic leukemia or in combination with a small-molecule inhibitor of Bruton’s kinase, ibrutinib or acalabrtinib, which have rendered conventional chemotherapeutic and immunotherapeutic approaches to being second tier approaches [69]. A key for defining the crosswalk from precision oncology targeting apoptosis to precision defined dietary/food patterns will be clearly delineating which regulatory node of apoptosis is misregulated in the specific molecular subtype of cancer that is being targeted. Targeting apoptosis is attractive because of the power of this process in reducing tumor burden [70]. This fact is evidenced in the use of a reduced dose of venetaclax at treatment initiation to ameliorate the risk of tumor lysis syndrome, i.e., a condition that can occur after treatment of fast-growing cancer, especially certain leukemias and lymphomas [71]. As tumor cells die, they break apart and release their contents into the blood. This causes a change in certain chemicals in the blood, which may cause damage to organs, including the kidneys, heart, and liver.

### 6.3. Angiogenesis

Angiogenesis is an essential process in the formation and development of tissues [72]. In the course of normal development, the arrangement of blood vessels becomes fixed, and angiogenesis is switched off. In adults, angiogenesis is only switched on during physiological processes such as wound healing or menstruation, and then only transiently, and it is regulated extremely carefully [72]. In contrast, cancer cells have angiogenesis constitutively switched on [73]. Many experiments have shown that proteins that block the action of key angiogenesis effectors, e.g., vascular endothelial growth factor (VEGF), are able to impair the growth of tumors; and this is now being exploited in the clinic [74]. Avastin was the first commercially available angiogenesis inhibitor: it binds directly to VEGF and prevents it from binding to the VEGF receptor [75]. Other recognized examples are endostatin, angiostatin, and thrombospondin-1 [76]. Small-molecule drugs that inhibit VEGF are in clinical trials, notably sorafenib, axitinib, and pazopanib [77]. The literature indicating that some common components of human diets also act as mild angiogenesis inhibitors exists as well [78,79,80]. Examples include foods in the botanical family Fabaceae (peas, beans, legumes), berries, green tea bioactives, and fungi, such as mushrooms.

### 6.4. Immune Checkpoints

Tumors evolve to avoid immune attacks. The tumor microenvironment is immunosuppressive [81]. PD-1, PD-L1, and CTLA-4 are the three most popular immune targets [82]. PD-1 is a member of the CD28 family and is an inhibitory receptor expressed on activated T cells, B cells, macrophages, regulatory T cells (Tregs), and natural killer (NK) cells. It has two binding ligands PD-L1 and PD-L2 expressed on normal cells. The combination of PD-1 with either of the ligands can inhibit T cell activity and induce T cell tolerance. Immunotherapy aims to inhibit the activity of these immune checkpoint blockers. Immunotherapy drugs called immune checkpoint inhibitors work by blocking checkpoint proteins from binding with their partner proteins [83]. They prevent the “off” signal from being sent, allowing the T cells to kill cancer cells. One such drug acts against a checkpoint protein called CTLA-4. So far, the six approved ICIs include (1) PD-1 inhibitors, such as pembrolizumab, nivolumab, and cemiplimab; (2) anti-PD-L1 inhibitors, including atezolizumab, and durvalumab; and (3) ipilimumab, an anti-CTLA-4 inhibitor (66). ICIs are the standard for the treatment of metastatic and unresectable, stage 3 non-small cell lung cancer.

The most significant benefit of immune checkpoint inhibitor therapy is to use the immune function to destroy tumors. The field initially focused on the development of antibodies to inactivate these molecular inhibitors of immunity, but the approach is expensive and associated with significant side effects. Consequently, the focus is shifting to small-molecule inhibitors of these proteins that are orally administered and can penetrate cell membranes to act in cells [84]. CA170 was the first to obtain a new drug research application for small-molecule immune checkpoint inhibitors [85]. CA-170 is the only small-molecule modulator that can be taken orally for PD-1 and VISTA pathways and is an immune activation negative checkpoint modulator. It is under clinical investigation (ClinicalTrials.gov, Identifier: NCT02812875, accessed on 1 February 2023). This is a rapidly emerging area because of the transformative nature of activation of the tumoricidal activity of the immune system at all stages of the carcinogenic process. The immunomodulatory potential of natural products used in herbal medicine has recently been reviewed [45].

## 7. Developing the Cross Walk between Precision Oncology and Precision Nutrition

Food-derived natural products, as defined above, provide a communications portal between precision oncology and nutrition, a hybrid approach that we now define as dietary oncopharmacognosy. Three well-developed and rapidly expanding resources will facilitate the effort to formulate precision dietary patterns: (1) natural products databases in the science of pharmacognosy (reviewed in [86]), (2) small-molecule drug development databases in which both successful and failed structural data reside [87], and (3) metabolomic databases of food components, also referred to as *foodomics* and often cataloged as part of the human exposome [88]. These resources, in combination with artificial intelligence-driven in silico experiments, can be used to define the first generation of precision-defined dietary/food patterns for clinical evaluation in Phase I trials. An approach similar to this was recently described to construct a food map with the anti-cancer potential of each ingredient defined by the number of cancer-beating molecules that the food contained [89].

## 8. Next Steps

**Figure 1:**
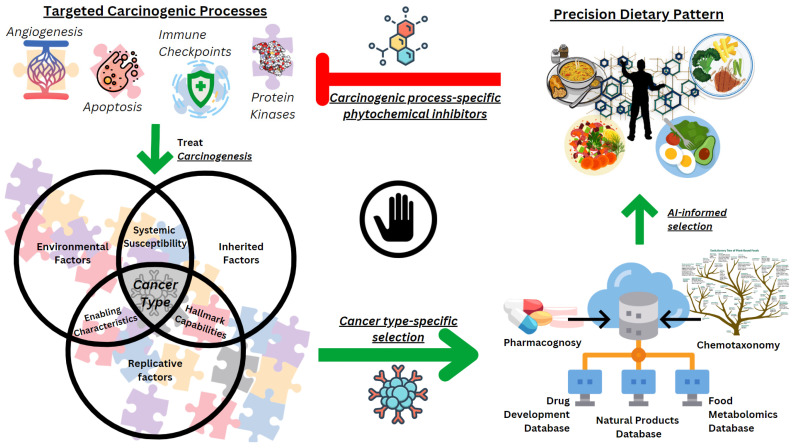
Dietary oncopharmacognosy. The figure emphasizes the intricate connection between the features of the carcinogenic process leading to the emergence of a particular type of cancer in a patient and the personalized approach to precisely adjust dietary patterns for the said patient to maximize prevention based on their cancer type. The Venn diagram (on the left) unifies theories on cancer development stating that a cancer cell gains growth and survival advantage over other cells owing to collective effects of external and internal influences, at the core of which, there are aberrant protein kinase signaling, enhanced angiogenesis, impeded apoptosis, and immune response breakout, which shift the stoichiometry of cellular homeostasis towards error-prone replication. The combination of these carcinogenic processes and the functional intensity of their impacts determine the fingerprint of a particular cancer type in a particular patient. However, available bioinformatic databases (on the right) provide information on which natural products and food metabolites contain active molecular structures that inhibit these specific carcinogenic processes, resembling the activity of drugs, and which groups of plants could synthesize them and be used as foods. This information can be refined using artificial intelligence technologies to tune specific dietary recommendations to deliver the combination of specific phytochemicals on a regular basis, resulting in corresponding collective inhibitory effects on the carcinogenic processes that drive that particular cancer type in a particular patient.

The conceptual framework outlined herein is shown in Figure 1. It must be subjected to critical proof-in-principle experimentation. In setting the stage for that work, we consider it irrefutable that transformative changes in cancer treatment have occurred due to the use of small-molecule inhibitors that target specific protein kinases and specific facets of apoptosis, angiogenesis, and immune checkpoint regulation [34,45,46]. In many cases, small-molecule inhibitors have replaced chemotherapy as the preferred treatment option [35,51]. Another aspect of the proposed framework that we consider beyond reproach is that the goal of prevention strategies, since the launch of the field of chemoprevention, is to reduce deaths from cancer by treating all stages of the carcinogenic process, not only the clinically detectable stage. More specifically, dietary pharmacognosy is designed not only to work hand-in-glove with cancer treatments that attack existing cancer cells but also to prevent malignant transformation and intercept undetected clones of neoplastic cells, i.e., the entire cancer continuum.

We judge that interest in the field of chemoprevention waned, because the early compounds tested lacked specificity, as did the molecular determinants that they were intended to target, particularly in comparison to the small molecules and their intended targets that are being evaluated today. Accordingly, rather than concluding that the concept of chemoprevention was a failure, we argue that the twenty-first-century successes in the use of small-molecule inhibitors in cancer treatment to reduce deaths from cancer demonstrate that chemoprevention works.

We make this point to argue that the same perceptions exist for current efforts to identify anti-oncogenic dietary/food patterns. It is also recognized that there will be a concern that bioactive chemicals in foods will lack the target specificity or affinity for the target in comparison to drugs in clinical use. There is no doubt that this argument is true. However, it is also important to recognize that many first-generation drugs are also less specific and of lower affinity than their second- and third-generation successors, and that the drug development efforts have sometimes argued that this is a benefit relative to the development of drug resistance [62,90]. In many respects, this concern can be reduced to stoichiometry between pro- and anti-cancer effector molecules with an assessment of whether the effects of precision food patterns are sufficient to delay death from cancer such that other disease process account for mortality. In view of these opportunities and in order to overcome recognized obstacles, we propose a series of the following steps.

1. ***Identify a specific molecular subtype of cancer.*** There are many molecular subtypes of cancer for which small-molecule drugs are in use. Serious consideration should be given to investigating cancer subtypes in which multiple cellular processes have been shown to be successfully targeted. This will increase the likelihood that precision food patterns will exert synergistic effects. It would also be beneficial for the selection of molecular cancer subtypes in which a watchful waiting period exists between initial diagnosis and initiation of treatment. Finally, consideration needs to be given to whether it is feasible to monitor the pharmacologic activity of food molecules systemically and on the target tumor cell population of the cancer subtype selected for investigation. Therefore, a particular cancer type in a particular patient provides a list of key identifying molecules, dysregulation of which continuously drives this carcinogenesis, and thus, they are to be targeted specifically in this patient.
2. ***Identify effective small-molecule inhibitors of that cancer and the pharmacokinetic and pharmacodynamic properties associated with successful outcomes.*** The drug development literature needs to be carefully mined for structure-activity relationships and to ensure that critical data about the pharmacological determinants of successful outcomes are available for examination. Standards for this purpose have been published, and the need to consider potential off-target effects has been emphasized [90,91].
3. ***Identify structural similarities in the identified small-molecule drugs and the natural products that are components of specific foods.*** The literature is replete with in silico analyses of structure relationships that have led to the identification and development of clinically effective small-molecule inhibitors. This has occurred in the science of pharmacognosy as well as in drug development [92]. These same approaches now need to be applied to the food metabolome: certain postprandial metabolites detected in circulation possess the same or functionally similar biochemical/pharmacological properties as the components of small-molecule drugs found in the previous step; therefore, exposure of tissues to them mimics administration of small-molecule inhibitors of interest, i.e., we are selecting for food metabolites of similar biochemical functionality as the small-molecule inhibitors selected for specific cancer type.
4. ***Design specific food patterns predicted to exert effects on identified targets.*** Some food metabolites can be found post-consumption in more than one type of food. For instance, in the realm of plant-based food patterns, there is a botanical classification method based on the similarity of phytochemicals found in a plant (*chemotaxonomy*) which can be utilized to determine the plants of interest providing specific food metabolites with the necessary inhibitory properties. This will allow diversifying the food patterns tailored for a specific patient based on their molecular cancer type, ensuring enough exposure to inhibitory phytochemicals to achieve desired long-term anti-cancer effects. Such precision food patterns that are to be developed must also satisfy guidelines, such as *Recommended Dietary Intakes* and *Dietary Guidelines for Americans*, considering the multi-faceted impact of the diet in determining the health status, especially considering the ongoing pandemic of metabolic disorders.
5. ***Assess effects in well-designed Phase I trials.*** Well-accepted trial designs have been established for the initial evaluation of small-molecule inhibitors that are now being successfully used in the clinic [93]. The trial protocols for precision dietary/food patterns should parallel those designs as food-derived metabolites with anti-cancer properties should be perceived as pharmacological agents based on the fact that they undergo similar kinetics and dynamics in an organism with a targeted effect of interest as any other pharmaceutical drugs (*food pharmacology*).

It is informative to note that studies of diet, nutrition, and cancer have given limited attention to hematological malignancies. On the other hand, a successful focus in drug development of small-molecule inhibitors has been on these same malignancies. For some hematological malignancies of high prevalence, such as chronic lymphocytic leukemia, there is frequently an extended period of watchful waiting between diagnosis and initiation of treatment. This situation provides a significant opportunity to evaluate food-based precision dietary patterns, where access to cancer cells is easily achieved, and the ability to perform pharmacokinetic and pharmacodynamic analyses are relatively straightforward, particularly in the context of proof-in-principle experiments that are required to assess the biological plausibility of dietary oncopharmacognosy.

## 9. Conclusions

In the mid-1970s, the conceptualization of the field of cancer chemoprevention was the source of great optimism. However, we argue that there was a shift in expectations for chemoprevention away from the prevention of death from cancer and that the compounds evaluated lacked target specificity. The many failures reported ultimately resulted in a loss of interest in chemoprevention. However, if one broadens perspective and returns to the original goal of chemoprevention to reduce deaths from cancer, the highly effective small-molecule inhibitors being used in the clinical treatment of cancer are twenty-first-century demonstrations that chemoprevention works because of a knowledge of various cellular processes that are dysregulated in carcinogenesis as specific targets of well-tolerated orally administered small-molecule inhibitors on the market or in active clinical trials. We now argue that demonstrating the role of diet and nutrition in cancer prevention and control has been similarly limited. Herein, we present a paradigm that reframes the approach to investigating precision dietary/food patterns for the reduction in cancer-related deaths and challenge the scientific community interested in this topic to refine further the said paradigm and to conduct proof-in-concept experiments based on an understanding of the pharmacokinetic and pharmacodynamic parameters that underlie the success of small-molecule inhibitors being used in cancer treatment. We refer to this precision approach as *dietary oncopharmacognosy* and envision it as the crosswalk between the currently defined fields of precision oncology and precision nutrition. We also emphasize that the challenges associated with such an approach, including but not limited to low potency of food metabolites, potential off-target effects due to multi-faceted functions thereof, relatively long time course to achieve the desired peak effect and maximum efficacy, are overshadowed by the magnitude of the synergistic effect from regular consumption of diverse foods containing multiple bioactive compounds targeting several nodes driving carcinogenesis in a particular person. We caution that in patients with already diagnosed cancer, such dietary oncopharmacognosy must supplement but not replace active cancer care and precision therapy so as to enhance the prevention of cancer-related death. However, proper diagnostics of individual cancer risk for a person based on their *a priori* predisposition for developing a certain type of cancer would ultimately maximize the benefits from dietary oncopharmacognosy that could potentially prevent carcinogenesis from occurring.

## Data Availability

Not applicable.
